# Push-Out Bond Strength of Dorifill, Epiphany and MTA-Fillapex Sealers to Root Canal Dentin with and without Smear Layer

**Published:** 2014-10-07

**Authors:** Mohammad Forough Reyhani, Negin Ghasemi, Saeed Rahimi, Amin Salem Milani, Hadi Mokhtari, Sahar Shakouie, Hossein Safarvand

**Affiliations:** a *Department of Endodontics, Dental School, Tabriz University of Medical Sciences, Tabriz, Iran;*; b *Dental and Periodontal Research Center, Dental School, Tabriz University of Medical Sciences, Tabriz, Iran;*; c *Private Practice, Tabriz, Iran*

**Keywords:** Dorifill, Epiphany Sealer, MTA-Fillapex, Push-Out Bond Strength, Root Canal, Smear Layer

## Abstract

**Introduction:** The aim of the present experimental study was to evaluate the push-out bond strength of Dorifill, Epiphany and MTA-Fillapex sealers to root canal dentin in presence and absence of smear layer (SL). **Methods and Materials:** Sixty human single-rooted teeth were selected and divided into six groups (*n=*10). The canal irrigation protocol in groups 1, 3 and 5 consisted of 2.5% NaOCl during instrumentation and normal saline at the end of preparation plus a 5-min irrigation with 17% ethylenediaminetetraacetic acid (EDTA). In the remaining groups, normal saline was used for canal irrigation. The root canals were filled with Epiphany/Resilon (groups 1 and 2), Dorifill/gutta-percha (groups 3 and 4) and MTA-Fillapex/gutta-percha (groups 5 and 6). After two weeks of storage in 95% relative humidity at 37^º^C, 2 mm-thick dentin disks were prepared from coronal third of each root. The push-out bond strength test was carried out using a universal testing machine. Data were analyzed with the two-way ANOVA and post hoc Tukey’s tests. Statistical significance was defined at 0.05. **Results: **The highest (3.06±0.38 MPa) and lowest (1.16±0.32 MPa) push-out bond strength values were recorded in Epiphany/Resilon-NaOCl/EDTA and Dorifill/gutta-percha/normal saline groups, respectively. There were significant differences in the bond strength of sealers (*P*<0.05). In addition, elimination of the SL significantly increased the bond strength of all sealers (*P*<0.05). **Conclusion:** The Epiphany/Resilon group exhibited the highest push-out bond strength in the presence and absence of the SL. Elimination of the SL resulted in a significant increase in the bond strength of all the sealers to dentin.

## Introduction

Adhesion to dentin is an essential property for root canal sealers [1-3]; and higher bond strength decreases leakage and improves the stability of root canal obturation material [1].

The adhesion properties of sealers to dentin might be different due to various reasons, including differences in root dentin structure between the samples or even between the different parts of the same sample, presence or absence of the smear layer (SL), the chemical composition of the sealer and its reaction with the dentin [4-6].

Various tests have been used to evaluate the bond strength, which include shear-bond strength, microtensile, pull-out and push-out tests [1]. Microtensile and push-out tests make it possible to measure the bond strength in different parts of the root canal and also to evaluate the differences in bonding in these different root segments. However, it is very difficult to prepare samples for microtensile test as they may fracture before the test. On the other hand, the push-out bond strength test does not have the limitations of microtensile test and therefore the results are more accurate and reliable [7-9].

One of the most important issues in evaluating the bond strength of materials to root canal dentin is the effect of SL on the adhesion of sealers. Based on the majority of reports, the bond strength to root dentin decreases in the presence of SL, irrespective of the type of the sealer [10]. A large number of sealers are being used with different chemical compositions, including zinc oxide-eugenol (ZOE)-based sealers such as Dorifill (DR; Dorident Company, Vienna, Austria) [11]. Resin sealers such as Epiphany (EP; Pentron Clinical Technologies, Wallingford, CT, USA) are from newer generations and are capable of forming a bond with dentin and the core material; the so-called mono-block [12, 13]. Recently, mineral trioxide aggregate (MTA)-based sealers have been introduced in order to achieve biologic properties and a proper seal with MTA. One of these sealers is MTA-Fillapex (MF; Angelus, Londrina, PR, Brazil) which is presented in the form of two pastes and apart from MTA, its chemical composition contains resins, bismuth oxide, silica nanoparticles and dyes. This sealer has high sealing ability, bactericidal effect and biocompatibility. Other properties of this sealer include radiopacity, low solubility and low setting expansion [14, 15].

Only a limited number of studies have evaluated the bonding ability of this sealer. Therefore, the aim of the present laboratory study was to evaluate the push-out bond strength of MF (MTA-based sealer), EP (resin-based sealer) and DR (ZOE-based sealer) to root dentin in the presence and absence of the SL.

## Methods and Materials

***Preparation of samples***

Sixty human maxillary central incisors were included in the present study. The inclusion criteria consisted of teeth with only one root canal, absence of previous root canal treatment, and absence of any carious lesions. All of the attached soft tissues were removed from the tooth surfaces with a periodontal curette (Hu-Friedy, Chicago, IL, USA) and the teeth were stored in 0.5% chloramine-T solution until the time of study.

The tooth crowns were removed at cementoenamel junction (CEJ) level using a diamond disk (SP 1600 Microtome, Leica, Nu Block, Germany) and the working length (WL) was determined at 1 mm short of the apical foramen with a #15 K-file (Dentsply Mailefer, Ballaigues, Switzerland). The root canals were prepared using the crown-down technique with RaCe rotary system as follows: #40/0.10 and # 35/0.08 for the coronal third, #30/0.06 for the middle third and #25/0.06 for preparation up to the WL.

The samples were divided into 6 groups based on the irrigation protocol and the root canal obturation material. In groups 1, 3 and 5 the irrigation protocol consisted of 2.5% NaOCl solution during instrumentation and a final flush with normal saline at the end of preparation procedures, followed by a 5-min use of 17% ethylenediaminetetraacetic acid (EDTA) (Pulpdent Corporation, Watertown, MA, USA). In the remaining groups, the irrigation was done with normal saline.

After preparation, the root canals were dried with paper points and obturated using lateral compaction technique, as follows: groups 1 (EP-RE 1) and 2 (EP-RE 2) with Epiphany sealer and Resilon points; groups 3 (DR-GP 3) and 4 (DR-GP 4) with gutta-percha and Dorifill sealer ; and groups 5 (MF-GP 5) and 6 (MF-GP 6) with gutta-percha and MTA-Fillapex sealer . In groups 1 and 2, before placing the Resilon points in the root canals, a primer (Epiphany Primer; Pentron Clinical Technologies LLC, Wallingford, CT, USA) was placed on the canal walls with a paper point according to the manufacturer’s instructions. Light-curing was carried out at canal orifices for 40 sec at the end of canal obturation. After completion of the obturation procedures, the quality of root canal filling was evaluated by radiographies.

The samples were stored at 95% relative humidity and 37^º^C. Then 2 mm-thick disks were prepared from the coronal third of root canals, using a diamond saw (SP 1600 Microtome; Leica, Nu Block, Germany).

***Push-out test***

Push-out test was carried out in a universal testing machine (Hounsfield Test Equipment, Model H5K-S, Surrey, England). The force was applied in the apico-cervical direction of the samples at a crosshead speed of 1 mm/min. The maximum force (F) applied at bond failure was recorded in Newton. The push-out bond strength was calculated in MPa according to the following formula: *δ*=F/2*πr*×*h* (with *π*=3.14, *r* being the radius of the root canal and *h* being the thickness of the disk sample in mm) [16].

***Statistical analysis***

The two-way ANOVA test was used to evaluate the significance of differences between the sealer type and irrigation protocol. The post hoc Tukey’s test was used for the two-by-two comparison of sealers’ resistance to displacement. The SPSS software (SPSS version 18.0, SPSS, Chicago, IL, USA) was used for data analysis and statistical significance was set at 0.05.

## Results

Descriptive analysis of data showed the highest and lowest bond strength values in group EP-RE 1 (NaOCl-EDTA) and group DR-GP 4 (normal saline), respectively (Table 1). The presence and absence of SL and the sealer type had a significant effect on bond strength (*P*=0.03 and 0.01, respectively). The EP-RE 1 and EP-RE 2 groups exhibited the highest resistance to displacement irrespective of the type of irrigation solution, whereas DR-GP 1 and DR-GP 2 groups showed the least resistance. Irrespective of the sealer type, the mean bond strength to dentin after irrigation with 2.5% NaOCl+17% EDTA was higher than irrigation with normal saline solution. 

**Table1:** Mean (SD) of bond strength values for different sealers (EP=Epiphany, MF=MTA-Fillapex, DR=Dorifill)

**Irrigation protocol**		**Mean (SD)**	
	**EP**	**MF**	**DR**
**2.5% NaOCl+17% EDTA**	3.06 (0.38)	1.98 (0.26)	1.26 (0.37)
**Normal saline**	2.03 (0.37)	1.59 (0.33)	1.16 (0.32)

The post hoc Tukey’s test showed significant differences between the three sealers (*P*=0.01), with higher mean bond strength belonging to EP compared to other two sealers and with higher bond strength with MF compared to DR sealer.

## Discussion

The present study compared the push-out bond strength of three root canal sealers to root dentin in absence and presence of the SL. The sealer type and the presence of the SL had significant effects on the bond strength. The bond strength values in descending order were recorded as EP, MF and DR groups and elimination of the SL with an irrigation protocol of 2.5% NaOCl+17% EDTA had a significant positive effect on increasing the resistance to displacement.

There are different techniques to measure the bond strength of materials and push-out test is one of the most reliable methods based on the results of previous studies. In this test the conditions are comparable to clinical conditions, in which the tested items are directly placed within prepared canals with normal tubular configuration and organization [17-19].

Adhesion to root dentin is one of the necessary characteristics of root canal sealers for two reasons: the superior seal which in turn results in less coronal and apical leakage [2], and preventing the displacement of the filling material during restorative procedures [18]. One of the factors affecting the adhesion characteristics of sealers is their chemical composition. The eugenol content of the ZOE-based sealers is chelated with the zinc oxide present in GP. In addition, this reaction can also take place with the calcium phase of dentin [6, 11]. Methacrylate-based resin sealers adhere to both the root dentin and the filling material, and this can effectively seal the root canal by penetration of resin tags into the dentinal tubules that bond with the collagen matrix [20, 21].

MTA-based sealers are based on resin salicylate and calcium silicate base. Considering the chemical composition, it is expected that there should be some similarities in bond strength to dentin between resin sealers and MTA-based sealers. Higher bond strength of this sealer compared to DR might be attributed to such similarities [22-24]. Moreover, Sarkar *et al*. [24] showed that release of calcium and hydroxyl ions from the set sealer results in the formation of apatite which comes into contact with fluids containing phosphate. Reyes-Carmona *et al.* [25] also reported that the apatite formed by MTA and phosphate salts, is deposited among collagen fibrils, resulting in a controlled increase in the formation of inorganic nucleations on the dentin, which are seen as an interfacial layer with tag-like structures. In this study the lower bond strength of MF, compared to EP, might be attributed to the lower adhesion capacity of these tag-like structures. This is consistent with the results reported by Nagas *et al*. [26] and Amin* et al*. [27].

In the present study, the EP-RE 1 and EP-RE 2 groups were more resistant to displacement compared to the DR-GP 3 and DR-GP 4 groups, which is in line with the results of studies by Sagsen *et al.* [28]*,* Pecora *et al. *[29] and Barbizam *et al. *[30]. Based on these studies, the higher bond strength of resin-based sealers (EP), compared to ZOE-based sealers (DR), can be attributed to the resin base that causes better and homogeneous penetration into tubules and bonding with the collagen matrix. Therefore, a higher mechanical retention is achieved between the sealer and root dentin, denoting better adhesion.

Another finding of the present study was the effect of the SL on bond strength. Based on the results of previous studies the most effective technique to remove the SL is the use of NaOCl and EDTA [31-35]. The results of the present study showed that irrespective of the type of the sealer used, the bond strength to dentin increased in all the groups with SL removal which is consistent with the results of the previous studies [1, 27, 32, 36, 37].

Electron microscopic evaluations have shown that removal of the SL results in the exposure of dentinal tubules and creation of an irregular surface [4]. Penetration of sealer into the dentinal tubules and surface irregularities improve the retention mechanism of sealers to root canal walls, especially with resin-based sealers [7, 33-35]. In this study the samples with SL exhibited the lowest bond strength values, which might be attributed to the presence of the SL on the surface of dentin. These findings emphasize that the presence of SL has a negative impact on the adhesion of sealer to dentin because it produces an interfacial layer between the sealer and dentin, which interferes with penetration of sealer into the dentinal tubules and formation of sealer tags; therefore, adhesion is compromised under micromechanical forces [38-40]. However, the findings reported by Gopikrishna *et al.* [41] on ZOE-based sealers are not consistent with these findings because they reported no significant differences in the bond strength of these sealers to dentin in the presence/absence of the SL, which might be attributed to the use of shear test instead of push-out test.

## Conclusion

The results of the present experimental study showed higher bond strength in the Epiphany/Resilon system compared to MTA-Fillapex and Dorifill. In addition, removal of the smear layer increased the resistance to displacement of root filling materials.
